# Comparing Relationship Satisfaction and Body-Image-Related Quality of Life in Pregnant Women with Planned and Unplanned Pregnancies

**DOI:** 10.3390/diseases12060109

**Published:** 2024-05-22

**Authors:** Razvan-Ionut Daniluc, Marius Craina, Barkha Rani Thakur, Mihaela Prodan, Melania Lavinia Bratu, Ana-Maria Cristina Daescu, George Puenea, Bogdan Niculescu, Rodica Anamaria Negrean

**Affiliations:** 1 Doctoral School, “Victor Babes” University of Medicine and Pharmacy Timisoara, 300041 Timisoara, Romania; razvan.daniluc@umft.ro (R.-I.D.); mihaela.prodan@umft.ro (M.P.); 2 Department of Obstetrics and Gynecology, “Victor Babes” University of Medicine and Pharmacy Timisoara, 300041 Timisoara, Romania; craina.marius@umft.ro; 3 Faculty of General Medicine, MediCiti Institute of Medical Sciences, Hyderabad 501401, India; barkharani0511@gmail.com; 4 Department of Plastic Surgery, “Victor Babes” University of Medicine and Pharmacy Timisoara, 300041 Timisoara, Romania; 5 Center for Neuropsychology and Behavioral Medicine, Discipline of Psychology, Faculty of General Medicine, “Victor Babes” University of Medicine and Pharmacy Timisoara, 300041 Timisoara, Romania; 6 Center for Cognitive Research in Neuropsychiatric Pathology, Department of Neurosciences, “Victor Babes” University of Medicine and Pharmacy Timisoara, 300041 Timisoara, Romania; 7 Department of Internal Medicine II, “Victor Babes” University of Medicine and Pharmacy Timisoara, 300041 Timisoara, Romania; ana-maria.daescu@umft.ro; 8 Department of Neurosciences, “Victor Babes” University of Medicine and Pharmacy Timisoara, 300041 Timisoara, Romania; 9 Department XVI, “Victor Babes” University of Medicine and Pharmacy Timisoara, 300041 Timisoara, Romania; puenea.george@umft.ro; 10 Department of Sports and Health, “Constantin Brancusi” University, 210152 Targu Jiu, Romania; bogdanniculescu78@yahoo.com; 11 Department of Preclinical Disciplines, Faculty of Medicine and Pharmacy, University of Oradea, 410073 Oradea, Romania; rodicanegrean@yahoo.com

**Keywords:** pregnancy, body image, relationship satisfaction, quality of life

## Abstract

This comparative cross-sectional study conducted at the “Pius Brinzeu” healthcare center in Timisoara explored the differential impacts of pregnancy planning status on sexual function, body image, and relationship satisfaction among pregnant women. Employing the Female Sexual Function Index (FSFI), Body Esteem Scale for Adolescents and Adults (BESAQ), and the Beck Depression Inventory (BDI-II), the study analyzed responses from 107 participants divided into groups of planned (*n* = 59, mean age 28.5 ± 5.2) and unplanned (*n* = 48, mean age 27.3 ± 4.8) pregnancies. In the first trimester, unplanned pregnancies reported higher median scores in desire (4.7 vs. 3.6, *p* = 0.005), arousal (4.5 vs. 3.8, *p* = 0.001), and lubrication (4.6 vs. 3.7, *p* = 0.015) compared to planned pregnancies. Satisfaction scores also favored unplanned pregnancies in the first trimester (4.8 vs. 3.9, *p* = 0.009). Similar trends were observed in subsequent trimesters, with unplanned pregnancies consistently reporting higher FSFI scores, indicating a robust sexual function. Risk factors significantly associated with sexual dysfunction were a higher BMI in the first trimester (beta coefficient: −0.124, *p* = 0.019), unmarried civil status (beta coefficient: −0.323, *p* = 0.045), history of previous abortion (beta coefficient: −0.451, *p* = 0.012), irregular menstrual cycles (beta coefficient: −0.384, *p* = 0.026), and rural living area (beta coefficient: −0.278, *p* = 0.034). Notably, unplanned pregnancy itself was not a significant risk factor for sexual dysfunction (beta coefficient: −0.054, *p* = 0.095). Regarding relationship dynamics, planned pregnancies exhibited significantly higher satisfaction with partner support (4.1 ± 0.9 vs. 3.7 ± 1.1, *p* = 0.041) and communication within the couple (4.0 ± 1.0 vs. 3.5 ± 1.2, *p* = 0.020), whereas unplanned pregnancies reported higher satisfaction with emotional closeness (4.3 ± 0.7 vs. 3.8 ± 1.0, *p* = 0.004). Concerns about managing professional activities and household chores were significantly more prevalent in the unplanned pregnancy group (62.50% vs. 33.90%, *p* = 0.014). Unplanned pregnancies demonstrated better initial sexual function but faced greater challenges in relationship satisfaction and managing pregnancy demands. Identifying and addressing the risk factors associated with sexual dysfunction can provide targeted interventions to improve the well-being of pregnant women, regardless of pregnancy planning status.

## 1. Introduction

The interplay between pregnancy planning status and its effects on women’s sexual and psychological well-being is a complex domain that has generated increasing academic interest [1,2,3]. Recent studies have underscored the significant psychological impacts of unplanned pregnancies, not only on maternal mental health but also on sexual function, relationship satisfaction, and body image perceptions, making them important for the holistic well-being of pregnant individuals, as they can have long-lasting effects on both the mother and the child [4,5,6].

Previous research has illustrated that pregnant women often experience changes in their sexual activity and satisfaction levels, which are influenced by physiological, psychological, and relational factors [7,8]. However, the dichotomy between planned and unplanned pregnancies adds an additional layer of complexity. Planned pregnancies are generally associated with positive anticipations and adjustments toward these changes, whereas unplanned pregnancies might pose challenges in adaptation, potentially exacerbating issues related to sexual dissatisfaction and body image concerns [9,10].

Furthermore, the role of relationship satisfaction in the context of pregnancy planning cannot be understated, as it serves as a critical buffer against the stressors associated with pregnancy, irrespective of its planning status [11,12]. The dynamics within a relationship, including emotional support and communication, play a significant role in navigating the changes and challenges during this period [13,14]. Other factors that are hypothesized to negatively influence relationship satisfaction are pregnancy complications, postpartum complications, and giving birth to children with congenital defects or genetic abnormalities, among others [15,16,17,18,19,20].

Recent studies across different countries have highlighted the complex nature of female sexual dysfunction (FSD). In Hungary, a neighboring country of Romania, with similar population characteristics, a survey categorized 20.3% of young women as experiencing FSD, with significant factors including sexual history and self-satisfaction (*n* = 5942) [21]. Slovenia, another Eastern European country, reported a 31% prevalence, with notable correlations between educational levels and various aspects of sexual function (*n* = 605) [22]. A global meta-analysis estimated the prevalence at around 50.75%, identifying depression, education level, and chronic diseases as key risk factors influencing sexual dysfunction in women of reproductive age [23].

This study hypothesizes that unplanned pregnancies are associated with lower sexual satisfaction, poorer body image, and decreased relationship satisfaction compared to planned pregnancies. The primary objective is to elucidate the differential impacts of pregnancy planning status on sexual function, body image, and relationship satisfaction among pregnant women.

## 2. Materials and Methods

### 2.1. Study Design and Ethical Considerations

This comparative cross-sectional study was conducted over a 12-month period, from December 2022 to December 2023. The research was based in the Obstetrics and Gynecology Department of “Pius Brinzeu” healthcare center, focusing on pregnant women who were receiving prenatal care within the facility and private clinics. The study aimed to compare relationship satisfaction and body-image-related quality of life between two groups of pregnant women: those with planned pregnancies and those with unplanned pregnancies. The assessment was facilitated through the online administration of the FFSFI and the BESAQ surveys.

All procedures performed in studies involving human participants were in accordance with the ethical standards of the institutional research committee and with the 1964 Helsinki Declaration and its later amendments or comparable ethical standards. The research protocol underwent a comprehensive review and received approval from the Ethical Committee for Scientific Research at the institution. The approval was documented under an assigned approval number, ensuring a clear and transparent ethical oversight process. Prior to participation in the study, informed consent was obtained from all individual participants involved in the research. This process included a detailed explanation of the study’s aims, methods, potential risks, and benefits, ensuring participants’ understanding and voluntary agreement to participate in the research. Confidentiality and anonymity of the participants were strictly maintained throughout the study, with data being handled in a manner that respects the privacy and dignity of all individuals involved.

The current methodology was structured around the PICO (Population, Intervention, Comparison, Outcome) framework. The Population of interest includes pregnant women across various stages of gestation, irrespective of age, socioeconomic status, and health background. The Intervention in this study is conceptualized as the occurrence of a planned pregnancy, where pregnancies were anticipated and prepared for by the women. The Comparison group consists of women who experienced unplanned pregnancies, where the pregnancies were not anticipated or prepared for at the time of conception. The Outcomes to be measured are twofold: relationship satisfaction and body-image-related quality of life, quantitatively assessed using two validated surveys: the FSFI and the BESAQ.

### 2.2. Inclusion and Exclusion Criteria

The inclusion criteria for participation in this study were (1) age between 18 and 45 years; (2) confirmed pregnancy; (3) ability and willingness to provide informed consent, ensuring ethical participation and understanding of the study’s scope and objectives; (4) capability to complete the FSFI and BESAQ surveys; and (5) fluency in the language in which the surveys were administered to guarantee accurate comprehension and completion of the questionnaires. Conversely, the exclusion criteria were defined to omit participants who might introduce confounding variables or whose participation could be ethically or medically inadvisable: (1) individuals with known cognitive impairments that could interfere with their ability to understand the informed consent or accurately complete the surveys; (2) pregnant women undergoing treatment for severe psychiatric conditions, as such conditions could independently affect their relationship satisfaction and body image, potentially confounding the study results; (3) those with high-risk pregnancies, including conditions like severe gestational hypertension or pre-eclampsia, to minimize health risks to the participants and their unborn children; and (4) any participant who chose to withdraw from the study at any stage, respecting each individual’s autonomy and right to discontinue participation without prejudice.

### 2.3. Analysis of Surveys

The Female Sexual Function Index (FSFI) and the Body Esteem Scale for Adolescents and Adults (BESAQ) are validated tools widely used to assess sexual function and body image, respectively [24,25]. The FSFI encompasses various dimensions of sexual function, including desire, arousal, lubrication, orgasm, satisfaction, and pain, providing a comprehensive overview of sexual health, with reported internal consistency ranging from 0.82 to 0.97 [26,27]. Conversely, the BESAQ assesses individuals’ feelings about their bodies, including weight concerns and physical condition, which are pivotal during the pregnancy and postpartum periods, having internal consistency scores ranging from 0.80 to 0.90 [28,29].

Besides an unstandardized questionnaire with 10 questions meant to assess satisfaction with current relationship and psychological status during pregnancy, the patients enrolled in the study were requested to fill in 3 standardized surveys. The FSFI is a self-administered survey designed to assess various aspects of sexual function in women. This 19-item questionnaire is widely recognized for its validity and reliability in measuring six key dimensions of sexual function: desire, arousal, lubrication, orgasm, satisfaction, and pain. Its utilization spans numerous studies, including psychometric research, investigations into sexual function and dysfunction, epidemiological surveys, and clinical trials focused on treatments for female sexual dysfunction (FSD). Scores on the FSFI range from 2 to 36, with a cutoff point of 26.55 or less serving as an indicator of potential FSD.

The BESAQ is a validated instrument comprising 28 questions that gauge an individual’s body image within sexual contexts. It evaluates the extent of self-consciousness, anxiety, and the tendency to avoid exposing certain physical attributes during sexual activities. The BESAQ has shown excellent psychometric qualities, making it a reliable tool for predicting sexual functioning. Scoring on this scale varies from 0 to 4, where lower scores denote greater comfort and less inhibition regarding body exposure in sexual scenarios, potentially leading to more enjoyable sexual experiences. Conversely, higher scores are indicative of increased anxiety and avoidance behaviors related to sexual engagement.

The BDI-II stands as one of the most esteemed instruments for assessing depressive symptoms across the globe. This 21-item self-report questionnaire delves into the common indicators of depression, including feelings of sadness, pessimism, guilt, loss of pleasure, thoughts of punishment, self-dislike, critical self-assessment, suicidal ideation, and physical symptoms such as changes in sleep, irritability, and fatigue. The BDI-II is known for its robust psychometric capabilities within both clinical and non-clinical populations. It features defined cutoff scores that categorize depression severity into four levels: normal (below 14), mild (14–19), moderate (20–28), and severe (29–63). The BDI-II internal consistency is above 0.90 [30].

### 2.4. Data Analysis

Sample size calculation was performed for a statistical power of 80%, a margin of error of 10%, and a 95% confidence interval. The population proportion for sample size calculation was considered at 20%, being the size of female population at reproductive age. A minimum of 62 cases was calculated as necessary, and a total of 150 women were invited to participate in the study. A total of 144 completed surveys were collected, and 107 were considered for the final analysis, after matching for age and background characteristics. Data management and analysis were conducted utilizing the statistical software SPSS version 26.0 (SPSS Inc., Chicago, IL, USA). Continuous variables were represented as mean ± standard deviation (SD), while categorical variables were expressed in terms of frequencies and percentages. Student’s *t*-test was used for comparing two means between the continuous data. The Chi-square test was utilized for the categorical variables. A *p*-value threshold of less than 0.05 was set for statistical significance. All results were double-checked to ensure accuracy and reliability.

## 3. Results

### 3.1. Background Analysis

The current study employed a total of 107 participants who successfully completed the given questionnaires and agreed to participate in the study, of which 59 had planned their pregnancy, compared with 48 who did not. In terms of age, the mean age for women with planned pregnancies was 28.5 years compared to the mean age of 27.3 years for those with unplanned pregnancies; however, this difference was not statistically significant (*p* = 0.257). A notable difference emerged in BMI measurements across the three trimesters of pregnancy. In the first trimester, women with unplanned pregnancies had a higher mean BMI (24.5) compared to those with planned pregnancies (23.1), with this difference being statistically significant (*p* = 0.019). The second trimester continued to show a statistically significant difference (*p* = 0.037), with unplanned pregnancies associated with a higher BMI (25.2) versus planned pregnancies (24.4). By the third trimester, while the mean BMI remained higher in the unplanned pregnancy group (26.1) compared to the planned pregnancy group (25.7), this difference was not statistically significant (*p* = 0.158).

The analysis further explored socioeconomic and lifestyle variables. Women with unplanned pregnancies were more likely to live in rural areas (52.08% vs. 33.90%, *p* = 0.034) and have a below-average income (39.58% vs. 25.42%, *p* = 0.073, approaching significance) and were more often unmarried (29.17% vs. 15.25%, *p* = 0.045). Moreover, a history of previous abortion was significantly more common among women with unplanned pregnancies (25% vs. 8.47%, *p* = 0.012), as was having an irregular menstrual cycle (35.42% vs. 18.64%, *p* = 0.026). In terms of contraceptive use, there was a significant difference between the two groups (*p* = 0.005), with a larger proportion of women who had unplanned pregnancies not using contraception (58.33%) compared to those with planned pregnancies (32.20%), as presented in Table 1.

### 3.2. Analysis of Unstandardized Surveys

A set of ten questions were addressed to all participants enrolled in the study to assess their relationship satisfaction. Regarding overall satisfaction with the current relationship, measured on a 1–5 scale, women with planned pregnancies reported a mean satisfaction level of 3.9 (±1.0), while those with unplanned pregnancies reported a slightly higher satisfaction level of 4.2 (±0.8), though this difference was not statistically significant (*p* = 0.094). Satisfaction with partner support differed significantly, with planned pregnancies reporting higher satisfaction (4.1 ± 0.9) compared to unplanned pregnancies (3.7 ± 1.1), with a *p*-value of 0.041, indicating that partner support satisfaction was higher among women with planned pregnancies.

Satisfaction with communication within the couple was higher among women with planned pregnancies (4.0 ± 1.0) compared to those with unplanned pregnancies (3.5 ± 1.2), with this difference being statistically significant (*p* = 0.020). Similarly, satisfaction with emotional closeness showed a significant difference, but interestingly, in favor of the unplanned pregnancy group (4.3 ± 0.7) compared to the planned pregnancy group (3.8 ± 1.0), with a *p*-value of 0.004.

The survey also explored adaptation to pregnancy and the ability to maintain daily activities amidst physical and psychological changes. There was no significant difference in adaptation to current pregnancy (88.14% vs. 81.25%, *p* = 0.158) or the ability to maintain daily activities due to physical changes (76.27% vs. 75.00%, *p* = 0.812) between the groups. However, the ability to maintain daily activities due to psychological changes from pregnancy was significantly higher in the planned pregnancy group (81.36%) compared to the unplanned pregnancy group (68.75%), with a *p*-value of 0.037.

Concerns about managing professional activities and household chores during pregnancy were significantly higher among the unplanned pregnancy group (62.50%) compared to the planned pregnancy group (33.90%), with a *p*-value of 0.014. Similarly, concerns about successfully carrying the pregnancy to term and worries about coping with labor and/or birth were significantly more prevalent among women with unplanned pregnancies, as described in Table 2.

### 3.3. Analysis of Standardized Surveys

In the first trimester, women with unplanned pregnancies reported significantly higher scores in the desire (median: 4.7 vs. 3.6, *p* = 0.005), arousal (median: 4.5 vs. 3.8, *p* = 0.001), and lubrication (median: 4.6 vs. 3.7, *p* = 0.015) domains compared to those with planned pregnancies. Although the median score for orgasm was higher in the unplanned pregnancy group (4.0 vs. 3.5), this difference was not statistically significant (*p* = 0.061). Satisfaction with sexual function was also significantly higher among women with unplanned pregnancies (median: 4.8 vs. 3.9, *p* = 0.009). There was no significant difference in pain scores between the two groups in the first trimester (*p* = 0.282). The total FSFI score was significantly higher in the unplanned pregnancy group (median: 29.1 vs. 26.5, *p* = 0.006), indicating better overall sexual function.

During the second trimester, significant differences persisted, though some domains like desire and orgasm did not reach statistical significance (*p* = 0.063 and *p* = 0.097, respectively). Arousal (median: 4.8 vs. 4.2, *p* = 0.019), lubrication (median: 5.0 vs. 4.3, *p* = 0.011), and satisfaction (median: 5.0 vs. 4.4, *p* = 0.008) scores remained significantly higher in the unplanned pregnancy group. No significant difference was observed in pain scores (*p* = 0.214). The total FSFI score again favored the unplanned pregnancy group (median: 28.2 vs. 25.4, *p* = 0.005).

In the third trimester, differences between the groups became somewhat more nuanced. Desire scores were significantly higher in the unplanned pregnancy group (median: 4.4 vs. 3.8, *p* = 0.039), as were lubrication (median: 4.9 vs. 4.2, *p* = 0.013), orgasm (median: 4.5 vs. 3.9, *p* = 0.025), and satisfaction (median: 5.0 vs. 4.3, *p* = 0.010) scores. There was no significant difference in arousal (*p* = 0.118) and pain (*p* = 0.186) scores between the two groups. The total FSFI score was significantly higher for the unplanned pregnancy group (median: 27.4 vs. 24.7, *p* = 0.007), as seen in Table 3.

In the planned pregnancy group, when comparing the first trimester with subsequent trimesters, there were no statistically significant changes in the median scores for desire, arousal, lubrication, orgasm, satisfaction, or pain (*p*-values ranged from 0.075 to 0.320). This suggests that sexual function in women with planned pregnancies remained relatively stable across the different stages of pregnancy. Specifically, the median total FSFI score showed a slight but not statistically significant decrease from the first trimester (26.5) to the third trimester (24.7), indicating a subtle decline in overall sexual function as the pregnancy progressed (*p* = 0.075).

**Figure 1:**
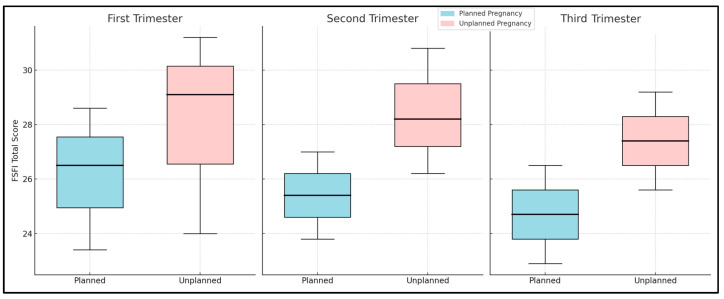
Trimester comparison of FSFI survey results.

Conversely, in the unplanned pregnancy group, similar trends were observed with no statistically significant changes in the scores for any of the sexual function domains across the trimesters (*p*-values ranged from 0.066 to 0.592). This group exhibited a slight decrease in the total FSFI score from the first trimester (29.1) to the third trimester (27.4), yet this change was not statistically significant (*p* = 0.133). Notably, the median scores for each domain generally started higher and remained higher throughout pregnancy compared to the planned pregnancy group, yet the slight decline in the total FSFI score suggests a modest reduction in overall sexual function over time (Table 4, Figure 1).

For the BESAQ scores, which reflect body esteem or positive feelings toward one’s body, there were no statistically significant differences between the planned and unplanned pregnancy groups across all three trimesters. In the first trimester, the median scores were 0.74 (IQR: 0.46–1.03) for the planned pregnancy group and 0.72 (IQR: 0.38–0.99) for the unplanned pregnancy group, with a *p*-value of 0.823. This pattern of similarity continued into the second trimester (*p* = 0.687) and the third trimester (*p* = 0.954), indicating that pregnancy planning status did not significantly affect body esteem levels among the participants at any point during pregnancy.

In contrast, the BDI scores, which assess the presence and severity of depressive symptoms, showed a different trend in the first trimester. Women with unplanned pregnancies exhibited higher median BDI scores (6.76, IQR: 3.24–9.62) compared to those with planned pregnancies (4.02, IQR: 3.38–6.42), and this difference was statistically significant (*p* = 0.037), suggesting higher levels of depressive symptoms in the unplanned pregnancy group during the first trimester. However, as pregnancy progressed into the second and third trimesters, the differences between the groups in median BDI scores decreased (*p* = 0.143 and *p* = 0.097, respectively), indicating that while the initial impact of pregnancy planning status on depressive symptoms was significant, it became less pronounced as pregnancy advanced (Table 5).

For the planned pregnancy group, the BESAQ scores, indicative of body esteem or positive body image, showed a slight increase from the first to the second trimester (from a median of 0.74 to 0.81) before experiencing a minor decline in the third trimester (to a median of 0.79). Despite these changes, the differences across trimesters were not statistically significant (*p* = 0.256), suggesting that body esteem remained relatively stable throughout pregnancy for women with planned pregnancies. Similarly, in the unplanned pregnancy group, the median BESAQ scores slightly increased from the first trimester (0.72) through to the third trimester (0.80), with no statistically significant change (*p* = 0.194), indicating a stable body-image-related quality of life across pregnancy stages.

The BDI scores, which measure depressive symptoms, for the planned pregnancy group started at a median of 4.02 in the first trimester, increased to 5.13 in the second trimester, and increased slightly further to 5.47 in the third trimester. However, these increases were not statistically significant (*p* = 0.312), suggesting that the level of depressive symptoms remained relatively consistent, with only a modest increase observed as the pregnancy progressed. For the unplanned pregnancy group, the median BDI scores began higher in the first trimester (6.76) compared to the planned group, decreased slightly in the second trimester (5.89), and then increased again in the third trimester (7.35), yet these fluctuations did not result in statistically significant changes (*p* = 0.257). This pattern suggests that while depressive symptoms varied throughout the pregnancy, the overall level of symptoms did not change significantly, as seen in Table 6.

### 3.4. Risk Analysis

BMI in the first trimester emerged as a significant factor for sexual dysfunction, as a dependent variable of the FSFI survey, with a beta coefficient of −0.124, suggesting that higher BMI values are associated with an increased risk of sexual dysfunction during pregnancy. Living in a rural area was also significantly associated with sexual dysfunction, with a beta coefficient of −0.278 and a *p*-value of 0.034. This could reflect limited access to healthcare services or differences in social and cultural attitudes toward pregnancy and sexual health in rural settings.

Unmarried civil status also showed a significant association, with a beta coefficient of −0.323 and a *p*-value of 0.045, indicating that unmarried women may face greater challenges related to sexual dysfunction during pregnancy. The history of previous abortion had a substantial negative impact, with a beta coefficient of −0.451 and a *p*-value of 0.012. This finding could point to psychological or physiological factors affecting sexual function following abortion experiences.

**Figure 2:**
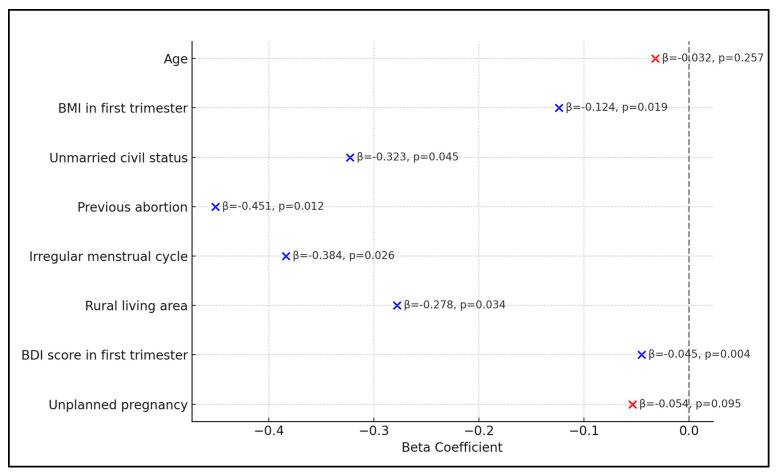
Risk factor analysis for sexual dysfunction (FSFI score as dependent variable).

Irregular menstrual cycles before pregnancy were significantly associated with sexual dysfunction, with a beta coefficient of −0.384 and a *p*-value of 0.026, potentially suggesting some underlying hormonal or reproductive health issues influencing sexual function. Lastly, unplanned pregnancies were not associated with a significantly higher risk of sexual dysfunction compared with women going through a planned pregnancy, with a beta coefficient of −0.054 (*p*-value = 0.095), as presented in Table 7 and Figure 2.

## 4. Discussion

### 4.1. Literature Findings

The current study found contrasting evidence from the initial hypothesis that women going through a planned pregnancy would have a better sexual and relationship life than those with an unplanned pregnancy. In reality, participants in the unplanned pregnancy group had significantly better scores in various domains. The nuanced findings from this study, particularly the higher levels of sexual function reported by women with unplanned pregnancies, invite a reconsideration of conventional wisdom regarding pregnancy, partnership, and sexual health. In contrast, the significant correlation between planned pregnancies and higher satisfaction with partner support and communication, juxtaposed with better sexual satisfaction among those with unplanned pregnancies, suggests that the dynamics of relationships and sexual experiences during pregnancy are more complex than previously understood.

The relationship between a higher BMI in the first trimester and an increased risk of sexual dysfunction presents a critical area for further exploration. This finding highlights the intersection of physical health and sexual well-being, suggesting that pregnancy-related body changes may influence sexual health in ways not entirely accounted for by current medical narratives.

Other findings regarding significant stress and concern over professional and household responsibilities among women with unplanned pregnancies raise important questions about the social and economic factors that shape pregnancy experiences. The diminishing difference in depressive symptoms as pregnancy progresses could indicate an adaptation process or the mobilization of support systems. The lack of statistically significant changes within each group across the trimesters suggests that while sexual function may vary individually, as a group, pregnant women experience relatively stable sexual function regardless of whether their pregnancy was planned or unplanned. However, the consistently higher scores in the unplanned pregnancy group across nearly all domains at all trimesters point to a potentially different sexual health dynamic in this group compared to those with planned pregnancies.

Moreover, the significant risk factors identified for sexual dysfunction, such as rural living area, unmarried civil status, and a history of previous abortion, underscore the socio-demographic dimensions of sexual health during pregnancy. These findings suggest that sexual dysfunction during pregnancy cannot be viewed solely through a biological lens but must be understood within a broader context of social support, healthcare access, and personal history.

In the context of unplanned pregnancies and the associated factors responsible for relationship well-being and sexual satisfaction, it is essential to study the background of the patients, from cultural, geographical, religious, and ethnical perspectives. Although data from Romania is not directly available, we can consider the latest studies that have reported an estimated rate of 54% of unplanned pregnancies in Eastern Europe, including Romania [31]. In this context, the studies by Wellings et al. [32] and Sarder et al. [33] provide valuable insights into the prevalence and determinants of unplanned and unintended pregnancies in distinct geographical contexts—Britain and South Asian countries, respectively. Wellings et al. found that 16.2% of pregnancies among women aged 16–44 years in Britain were unplanned, with an annual prevalence estimate of 1.5%. The factors strongly associated with unplanned pregnancy included first sexual intercourse before 16 years of age, current smoking, recent use of drugs other than cannabis, and lower educational attainment. Conversely, Sarder et al. reported that 19.1% of pregnancies across six South Asian countries were unintended, with notable variations from 11.9% in India to 28.4% in Bangladesh. Key determinants of unintended pregnancies included being younger, poorer, living in urban areas, having no children or a smaller family, the intention to use contraceptives, living with a partner, and early cohabitation.

In the context of exploring relationship satisfaction and body-image-related quality of life among pregnant women with varying pregnancy intentions, findings from Daescu et al. [7,34] reveal a complex landscape where hormonal imbalances, body image perceptions, and sexual function significantly intersect. Women with Polycystic Ovary Syndrome (PCOS) exhibit a more than 30% higher risk of sexual dysfunction, with elevated BESAQ scores, indicative of body image concerns, correlating with lower FFSFI scores. This association underscores the profound impact of body image on sexual health, beyond the physiological implications of PCOS. It was also observed that higher BESAQ scores increase the risk of female sexual dysfunction by 4.24 times. Similarly, hormones such as testosterone and the LH/FSH ratio were found to independently predict FSD. Moreover, during pregnancy, sexual function metrics such as lubrication, satisfaction, and pain experiences deteriorate, further influenced by psychological factors like depression (with increasing Beck’s Depression Inventory scores) and the nuances of relationship satisfaction. Notably, relationship satisfaction emerged as an independent predictor of sexual dysfunction, affirming the critical role of interpersonal dynamics in navigating sexual health and body image during pregnancy.

Other studies, when viewed through the lens of investigating relationship satisfaction and body-image-related quality of life in pregnant women with planned and unplanned pregnancies, accentuate the intricate links between pregnancy intentions, sexual function, and emotional well-being. Higgins et al. [35] uncover the complex emotional and relational dynamics fueling pregnancy ambivalence, which may lead to poorer contraceptive use and, consequently, unplanned pregnancies. Meanwhile, Koolaee et al. [36] observe that women with unintended pregnancies report significantly lower levels of sexual function and higher instances of physical and sexual abuse, alongside symptoms of anxiety and depression, affecting their overall life satisfaction. These insights collectively highlight the profound impact of pregnancy intentionality on women’s sexual and psychological health. They suggest that addressing sexual function and emotional well-being in the context of pregnancy requires a nuanced understanding of the interplay between relationship dynamics, body image concerns, and the broader social and emotional contexts in which pregnancies occur, pointing toward the necessity for holistic healthcare approaches that cater to these multifaceted needs.

Moreover, findings from Barrow et al. [37] and Gianotten et al. [38] collectively highlight the complex interplay between pregnancy intentionality, sexual satisfaction, and the broader socio-demographic and relational factors affecting reproductive health. Barrow et al. identify a significant prevalence of unplanned pregnancy (25.3%) in The Gambia, with variations across age, marital status, and number of children, underscoring the importance of tailored sex education and family planning services. Gianotten et al., in contrast, delve into the emotional and psychological challenges couples face when conception takes longer than expected, linking this struggle to decreased sexual desire and conception inefficiency and advocating for the inclusion of sexuality in pre-conception care to prevent sexual disturbances. These studies together underscore the necessity of addressing reproductive health through a multifaceted lens that not only focuses on preventing unplanned pregnancies through enhanced public health initiatives but also on nurturing sexual and relational well-being among couples striving for conception, pointing toward a holistic approach in reproductive and sexual healthcare provision.

### 4.2. Limitations and Future Perspectives

Several limitations are worth mentioning, such as the reliance on self-reported measures which introduces potential bias, as participants’ responses could be influenced by their current emotional state or societal expectations regarding pregnancy. Furthermore, the cross-sectional design restricts our ability to draw causal inferences from the observed associations, making it challenging to determine the directionality of these relationships. Additionally, the sample size, while sufficient for detecting significant differences, may not fully capture the diversity of experiences across different cultural, socioeconomic, and geographical backgrounds, limiting the generalizability of the findings. To enhance the study’s findings, future research should consider employing longitudinal designs, incorporating qualitative methods to offset the biases of self-reporting, and expanding the demographic reach to better capture cultural and socioeconomic diversity.

## 5. Conclusions

The findings from this study elucidate the complex interplay between pregnancy planning status and its implications on sexual function, relationship satisfaction, and risk factors for sexual dysfunction among pregnant women. The data indicate that while unplanned pregnancies may initially experience better sexual function, they also face significant challenges in relationship dynamics, particularly in partner support and communication. These insights highlight the necessity for healthcare providers to offer comprehensive prenatal support that addresses not only the physical aspects of pregnancy but also the emotional and relational dimensions. By recognizing and managing the identified risk factors for sexual dysfunction, such as a high BMI, unmarried status, previous abortions, irregular menstrual cycles, and rural residency, targeted interventions can be developed to support all pregnant women effectively. This approach can help in enhancing the overall well-being and satisfaction of pregnant women, thereby contributing to healthier pregnancy outcomes.

## Figures and Tables

**Table 1:** Comparison of background characteristics.

Variables	Planned Pregnancy (*n* = 59)	Unplanned Pregnancy (*n* = 48)	*p*-Value
Age (mean ± SD)	28.5 ± 5.2	27.3 ± 4.8	0.257
Level of education, *n* (%)			0.082
Elementary	12 (20.34%)	15 (31.25%)	
Mid-level	22 (37.29%)	20 (41.67%)	
Higher education	25 (42.37%)	13 (27.08%)	
BMI (mean ± SD)			
First trimester	23.1 ± 2.9	24.5 ± 3.2	0.019
Second trimester	24.4 ± 3.1	25.2 ± 3.5	0.037
Third trimester	25.7 ± 3.4	26.1 ± 3.6	0.158
Unemployed, *n* (%)	8 (13.56%)	10 (20.83%)	0.215
Below-average income, *n* (%)	15 (25.42%)	19 (39.58%)	0.073
Rural living area, *n* (%)	20 (33.90%)	25 (52.08%)	0.034
Unmarried civil status, *n* (%)	9 (15.25%)	14 (29.17%)	0.045
Previous abortion, *n* (%)	5 (8.47%)	12 (25.00%)	0.012
Irregular menstrual cycle, *n* (%)	11 (18.64%)	17 (35.42%)	0.026
Contraceptive use, *n* (%)			0.005
No	19 (32.20%)	28 (58.33%)	
<1 year	15 (25.42%)	8 (16.67%)	
1–5 years	20 (33.90%)	10 (20.83%)	
>5 years	5 (8.48%)	2 (4.17%)	

SD—Standard Deviation.

**Table 2:** Comparison of unstandardized survey results.

Variables (Mean ± SD)	Planned Pregnancy (*n* = 59)	Unplanned Pregnancy (*n* = 48)	*p*-Value
Overall satisfaction with current relationship (1–5 scale)	3.9 ± 1.0	4.2 ± 0.8	0.094
Satisfaction with partner support (1–5 scale)	4.1 ± 0.9	3.7 ± 1.1	0.041
Satisfaction with communication in couple (1–5 scale)	4.0 ± 1.0	3.5 ± 1.2	0.020
Satisfaction with emotional closeness (1–5 scale)	3.8 ± 1.0	4.3 ± 0.7	0.004
Satisfaction with current pregnancy adaptation (yes, %)	88.14% (52)	81.25% (39)	0.158
Ability to maintain daily activities due to physical changes from pregnancy (yes, %)	76.27% (45)	75.00% (36)	0.812
Ability to maintain daily activities due to psychological changes from pregnancy (yes, %)	81.36% (48)	68.75% (33)	0.037
Concerns about managing professional activities and household chores during pregnancy (yes, %)	33.90% (20)	62.50% (30)	0.014
Concerns about successfully carrying pregnancy to term (yes, %)	25.42% (15)	58.33% (28)	0.003
Worries about coping with labor and/or birth (yes, %)	30.51% (18)	56.25% (27)	0.001

SD—Standard Deviation.

**Table 3:** Comparison of FSFI survey results between planned and unplanned pregnancies.

Variables (Median-IQR)	Planned Pregnancy (*n* = 59)	Unplanned Pregnancy (*n* = 48)	*p*-Value
First trimester			
Desire	3.6 (2.8–4.4)	4.7 (3.6–5.8)	0.005
Arousal	3.8 (3.1–4.3)	4.5 (4.0–5.3)	0.001
Lubrication	3.7 (2.9–4.5)	4.6 (4.1–5.1)	0.015
Orgasm	3.5 (2.7–4.3)	4.0 (3.4–4.8)	0.061
Satisfaction	3.9 (3.1–4.7)	4.8 (4.2–5.2)	0.009
Pain	4.0 (3.2–4.8)	3.9 (3.3–5.3)	0.282
Total	26.5 (23.4–28.6)	29.1 (24.0–31.2)	0.006
Second trimester			
Desire	4.0 (3.4–4.6)	4.5 (4.1–4.9)	0.063
Arousal	4.2 (3.6–4.7)	4.8 (4.3–5.3)	0.019
Lubrication	4.3 (3.7–4.9)	5.0 (4.5–5.5)	0.011
Orgasm	4.0 (3.4–4.8)	4.6 (4.1–5.1)	0.097
Satisfaction	4.4 (3.8–4.9)	5.0 (4.6–5.4)	0.008
Pain	4.5 (3.9–5.1)	4.1 (3.6–5.6)	0.214
Total	25.4 (23.8–27.0)	28.2 (26.2–30.8)	0.005
Third trimester			
Desire	3.8 (3.2–4.4)	4.4 (4.0–4.8)	0.039
Arousal	4.1 (3.5–4.7)	4.3 (3.8–5.2)	0.118
Lubrication	4.2 (3.6–4.8)	4.9 (3.9–5.4)	0.013
Orgasm	3.9 (3.3–4.5)	4.5 (4.0–5.0)	0.025
Satisfaction	4.3 (3.7–4.9)	5.0 (4.5–5.3)	0.010
Pain	4.4 (3.8–5.0)	4.6 (3.5–5.5)	0.186
Total	24.7 (22.9–26.5)	27.4 (25.6–29.2)	0.007

IQR—Interquartile Range; FSFI—Female Sexual Function Index (higher scores indicate better sexual function).

**Table 4:** Trimester comparison of FSFI survey results.

Variables (Median-IQR)	Planned Pregnancy Group			*p*-Value	Unplanned Pregnancy Group			*p*-Value
	First Trimester	Second Trimester	Third Trimester		First Trimester	Second Trimester	Third Trimester	
Desire	3.6 (2.8–4.4)	4.0 (3.4–4.6)	3.8 (3.2–4.4)	0.286	4.7 (3.6–5.8)	4.5 (4.1–4.9)	4.4 (4.0–4.8)	0.080
Arousal	3.8 (3.1–4.3)	4.2 (3.6–4.7)	4.1 (3.5–4.7)	0.091	4.5 (4.0–5.3)	4.8 (4.3–5.3)	4.3 (3.8–5.2)	0.219
Lubrication	3.7 (2.9–4.5)	4.3 (3.7–4.9)	4.2 (3.6–4.8)	0.133	4.6 (4.1–5.1)	5.0 (4.5–5.5)	4.9 (3.9–5.4)	0.126
Orgasm	3.5 (2.7–4.3)	4.0 (3.4–4.8)	3.9 (3.3–4.5)	0.320	4.0 (3.4–4.8)	4.6 (4.1–5.1)	4.5 (4.0–5.0)	0.183
Satisfaction	3.9 (3.1–4.7)	4.4 (3.8–4.9)	4.3 (3.7–4.9)	0.308	4.8 (4.2–5.2)	5.0 (4.6–5.4)	5.0 (4.5–5.3)	0.592
Pain	4.0 (3.2–4.8)	4.5 (3.9–5.1)	4.4 (3.8–5.0)	0.126	3.9 (3.3–5.3)	4.1 (3.6–5.6)	4.6 (3.5–5.5)	0.066
Total	26.5 (23.4–28.6)	25.4 (23.8–27.0)	24.7 (22.9–26.5)	0.075	29.1 (24.0–31.2)	28.2 (26.2–30.8)	27.4 (25.6–29.2)	0.133

IQR—Interquartile Range; FSFI—Female Sexual Function Index.

**Table 5:** Comparison of BESAQ and BDI survey results.

Variables (Median-IQR)	Planned Pregnancy (*n* = 59)	Unplanned Pregnancy (*n* = 48)	*p*-Value
BESAQ			
First trimester	0.74 (0.46–1.03)	0.72 (0.38–0.99)	0.823
Second trimester	0.81 (0.52–1.19)	0.77 (0.39–1.05)	0.687
Third trimester	0.79 (0.41–1.07)	0.80 (0.32–1.08)	0.954
BDI			
First trimester	4.02 (3.38–6.42)	6.76 (3.24–9.62)	0.037
Second trimester	5.13 (2.88–7.34)	5.89 (4.22–9.11)	0.143
Third trimester	6.47 (3.19–8.56)	7.35 (4.68–10.29)	0.097

IQR—Interquartile Range; BESAQ—Female Sexual Function Index (higher scores indicate higher levels of body esteem or more positive feelings); BDI—Beck’s Depression Inventory (higher scores indicate greater depressive symptoms).

**Table 6:** Trimester comparison of BESAQ and BDI survey results.

Variables (Median-IQR)	Planned Pregnancy Group			*p*-Value	Unplanned Pregnancy Group			*p*-Value
	First Trimester	Second Trimester	Third Trimester		First Trimester	Second Trimester	Third Trimester	
BESAQ	0.74 (0.46–1.03)	0.81 (0.52–1.19)	0.79 (0.41–1.07)	0.256	0.72 (0.38–0.99)	0.77 (0.39–1.05)	0.80 (0.32–1.08)	0.194
BDI	4.02 (3.38–6.42)	5.13 (2.88–7.34)	5.47 (3.19–8.56)	0.312	6.76 (3.24–9.62)	5.89 (4.22–9.11)	7.35 (4.68–10.29)	0.257

IQR—Interquartile Range; BESAQ—Female Sexual Function Index; BDI—Beck’s Depression Inventory.

**Table 7:** Risk factor analysis (multiple linear regression) for sexual dysfunction based on the FSFI questionnaire.

Risk Factors *	Beta Coefficient	95% CI	*p*-Value
Age	−0.032	(−0.072, 0.008)	0.257
BMI in first trimester	−0.124	(−0.194, −0.054)	0.019
Unmarried civil status	−0.323	(−0.519, −0.127)	0.045
Previous abortion	−0.451	(−0.697, −0.205)	0.012
Irregular menstrual cycle	−0.384	(−0.598, −0.170)	0.026
Rural living area	−0.278	(−0.442, −0.114)	0.034
BDI score in first trimester	−0.045	(−0.075, −0.015)	0.004
Unplanned pregnancy	−0.054	(−0.086, −0.022)	0.095

*—Lower values are representative for sexual dysfunction; BMI—Body Mass Index; BDI—Beck Depression Inventory.

## Data Availability

The original contributions presented in the study are included in the article, further inquiries can be directed to the corresponding author.
